# Fast half-sibling population reconstruction: theory and algorithms

**DOI:** 10.1186/1748-7188-8-20

**Published:** 2013-07-12

**Authors:** Daniel Dexter, Daniel G Brown

**Affiliations:** 1 David R Cheriton School of Computer Science, University of Waterloo, Waterloo, ON N2L 3G1, Canada

**Keywords:** Kinship discovery, Half-sibling, Population genetics, Conservation biology, Heuristics

## Abstract

**Background:**

Kinship inference is the task of identifying genealogically related individuals. Kinship information is important for determining mating structures, notably in endangered populations. Although many solutions exist for reconstructing full sibling relationships, few exist for half-siblings.

**Results:**

We consider the problem of determining whether a proposed half-sibling population reconstruction is valid under Mendelian inheritance assumptions. We show that this problem is *NP*-complete and provide a 0/1 integer program that identifies the minimum number of individuals that must be removed from a population in order for the reconstruction to become valid. We also present SibJoin, a heuristic-based clustering approach based on Mendelian genetics, which is strikingly fast. The software is available at http://github.com/ddexter/SibJoin.git+.

**Conclusions:**

Our SibJoin algorithm is reasonably accurate and thousands of times faster than existing algorithms. The heuristic is used to infer a half-sibling structure for a population which was, until recently, too large to evaluate.

## Background

Conservation biologists and molecular ecologists use pedigree analysis to gain insight into the mating habits of populations. For example, knowing the reproduction mechanics of a population helps biologists make important ecological decisions about a region [1,2]. The information may also be used to assist in reproduction and conservation of endangered or threatened species [3,4]. A sub-field of pedigree analysis focus on relationships among same-generation individuals. Identifying related full sibling individuals, or individuals who share both a common mother and common father, is well studied and many algorithms exist for inferring relationships in such populations [5]. A similar, but much more difficult, task involves discovering individuals who are related by a single parent, also known as half-siblings. The ability to infer half-sibling relationships extends to being able to understand full-sibling relationships, but the converse of this is not true. Correct half-sibling reconstruction also allows biologists to develop a more complete understanding of how species mate than is possible with full-sibling reconstruction alone.

Knowing half-sibling relationships has important real-world applications and answers questions that full sibling reconstruction cannot. For example, knowing which individuals share a single common parent allows biologists to measure the degree of polygamy within a population [6]. Half-sibling reconstruction gives insight about pollination patterns, as mothers are pollinated by potentially distant fathers. The diversity of pollinators can be used to measure the degree of isolation, due to deforestation, which threatens many forests [1].

For diploid species, children inherit one maternal and one paternal chromosome at each locus from their parents. Mendelian genetic properties can identify unrelated individuals, but they also allow us to make predictions about related individuals. Unfortunately, we show that, unlike for full-siblings, determining whether a proposed half-sibling relationship structure obeys Mendelian inheritance rules is *NP*-complete.

The *NP*-hardness result indicates that any algorithm that attempts to provide valid Mendelian family relationships to polygamous populations will likely require a running time that is exponential in the size of the population, unless the objective being optimized is trivial. However, we provide an extremely fast heuristic, called SibJoin, which creates reasonably accurate population reconstructions in polynomial time. We also describe a 0/1 integer program that identifies the minimum number of individuals that must be removed in order to make a proposed population reconstruction valid under Mendelian inheritance rules. SibJoin uses the principle that if the genotypes of two individuals are very similar, we can be more confident that they are related than we can of two individuals with much different genotypes. The accuracy and speed of our algorithm allows us to infer half-sibling relationships for previously inaccessible population sizes. We reconstruct half-sibship partitionings for a real population of 672 kelp rockfish that previous half-sibling reconstruction algorithms fail to solve [7]. SibJoin is written in Python 2.7 and may be checked out from the master branch of its git repository at http://github.com/ddexter/SibJoin.git+.

### Related work

Many groups have produced algorithms for constructing full-sibling partitions. Current sibling reconstruction techniques fall into three categories: likelihood estimation, combinatorial objective optimization, and heuristics. While full sibling reconstruction is a well studied problem with many very accurate algorithms, half-sibling reconstructions algorithms are relatively few.

#### *Likelihood estimation*

Likelihood methods estimate the probability of the data under different partitionings of a population. An optimal solution maximizes this probability. For population reconstruction these strategies are often very slow, even with local search heuristics, which makes them ill suited for sibling reconstruction on large data sets. On the other hand, because this class of algorithm establishes a probabilistic model, it is often possible to directly incorporate error handling and prior assumptions about the population to increase accuracy. For a more detailed discussion of likelihood methods, see Jones and Wang [5].

COLONY [8] and COLONY 2.0 [9] are likelihood methods which construct half-sibling families. COLONY reconstructs full sibling families with high accuracy, but allows for polygamy in only one sex. COLONY 2.0 performs half sibship reconstruction when both sexes are polygamous. Both of these programs use a likelihood function and simulated annealing to find an optimal sibling structure for a population. However, results by Sheikh *et al.*[7], as well as our own results, show that COLONY and COLONY 2.0 become prohibitively slow for even medium-sized populations. Additionally, as demonstrated in Almudevar and Anderson [10], COLONY 2.0 often splits true sibgroups into smaller groups, leading to an incomplete reconstruction.

#### *Combinatorial optimization*

Combinatorial optimization solutions seek to provide a sibship partitioning which minimizes or maximizes some objective function, such as number of families, matings, or parents. As with likelihood methods, finding global optima for large populations can be computationally demanding. However, many optimization techniques are easily parallelizable.

KINALYZER [11] seeks a minimum set cover, by using an integer programming (IP) formulation where each set is subject to the restrictions of Mendelian compatibility for full siblings. KINALYZER yields decent results [12]; however, like the COLONY programs, does not scale well with population size. The minimum set cover objective used by KINALYZER is *NP*-hard [12]. Recent work has included half-sibling IP strategies that are similar to the full sibling strategies in KINALYZER, but they are unsuccessful for large populations [7]. The most viable of these is the half-sibling minimum set cover (HS-MSC) IP. Both COLONY and the HS-MSC cannot estimate half-sibling groups for large populations due to slow runtimes. Additionally, there is no evidence that minimizing the number of sibgroups is the right thing to do in all instances [10].

#### *Fast heuristics*

By making simplifying observations, heuristics can produce reasonably accurate results thousands of times faster than pure likelihood or combinatorial methods. Brown and Berger-Wolf propose a clustering algorithm which joins two individuals based on the number of genetically compatible third parties [13]. The assumption is, if two individuals form a large number of compatible full sibling triplets, then they are likely to be full siblings, alongside the recognition that any incompatible proposed family includes an incompatible triplet, which Brown and Berger-Wolf also prove. For a population of *n* individuals with *m* loci, this algorithm has an *O*(*n*^3^*m*) runtime and gives accurate results for modest numbers of alleles and loci.

Another heuristic, employed in PRT 2 [10], enumerates a list of maximal sibgroups: sibgroups for which no additional population may be added. PRT makes the assumption that it is unlikely to find unrelated individuals in a large sibgroup of this form. A set cover of the maximal sibgroups is then selected using a likelihood function. Although the authors claim that PRT supports half-siblings, half-sibling groups are never directly computed. Instead, full sibling groups are presented with a list of which pairs of groups can form valid half-siblings. This is problematic in instances where both sexes are highly polygamous because there will be many pairs of full sibling families that are also half-sibling compatible, and PRT does not indicate which of these are true half-sibling groups nor divide the half-sibling groups into the maternal and paternal groups. In fact, determining valid half-sibling families is *NP*-complete, as we show below, though for small problem instances or special cases, this may not be a major concern.

### Notation

Information about individuals’ genotypes are collected and expressed through the measurement of *microsatellites*, sequences of repeating DNA base pairs, such as ATATATAT, on a chromosome. The number of repeats gives an integer value denoting the *allele* for an individual. Microsatellites are collected from both chromosomes, though it is impossible to distinguish the two chromosomes with inexpensive technology. Each measurement site is called a microsatellite *locus*. In practice, scientists identify and report alleles at multiple loci in a population, typically to a maximum of one locus on each autosomal chromosome, to avoid linkage effects and recombination.

SibJoin requires that each individual be diploid, meaning that population members possess two of each type of chromosome. Exactly one chromosome is inherited from each of the individual’s parents; therefore, each locus will have a maternal and paternal allele. Let *m* be the number of measured loci for a population. Each locus will have a variable number of alleles, *k*, which we represent as *A*_*l*_ = {*a*_0_,*a*_1_,…,*a*_*k*−1_}.

When the inherited maternal and paternal alleles are combined, they give an individual’s genotype, which is unordered: (*a*_*i*_,*a*_*j*_) is equivalent to (*a*_*j*_,*a*_*i*_). Unfortunately, it is not always possible to reconstruct an individual’s alleles for a given locus. *Allelic dropout* is a term that refers to a common error in genotyping where information about a locus cannot be confidently determined and is omitted. We express sites with allelic dropouts as (∗,∗). When the same allele is inherited from both parents, the genotype is *homozygous*; when they differ, it is *heterozygous*.

The half-sibling problem is, given a population of *n* offspring, to reconstruct a maternal and paternal partitioning ℳ and P of the population that are consistent with Mendelian laws. for each maternal half-sibgroup M∈ℳ and for each paternal half-sibgroup P∈P, there must be a genotype for that sibgroup such that the individuals in *F* := *M*∩*P* must be valid offspring of those two genotypes. Further, the genotype chosen for a group *M* or *P* must be the same in all families that derive from that common parent. To avoid triviality, we typically seek to optimize some objective function when choosing the two partitions, as otherwise, one could simply assign each individual to a unique pair of parents. Our heuristic SibJoin relies on measurements of similarity between individuals. We denote the similarity between individuals *x* and *y* as *s*_*xy*_ and the similarity between clusters *C*_*i*_ and *C*_*j*_ as *sim*(*C*_*i*_,*C*_*j*_).

### Mendelian compatibility

Berger-Wolf *et al.*[14] give two Mendelian properties of diploid full siblings. Refer to their article for the concrete mathematical expression; here, we give a short exegesis. In any full sibgroup, at all alleles, at most four alleles appear since there are two parents each with at most two alleles. Berger-Wolf *et al.* refer to this rule as the 4-allele property. The 2-allele property enforces the rule that for each full sibling group, there is a partitioning of the alleles at each locus into a maternal and paternal group, such that each individual obtains exactly one allele from the maternal set and one from the paternal set, at each locus. Sheikh *et al.*[7] extend these rules to half-siblings. The *half-sibship property* states that for each locus in a half-sibling family, there exists two alleles {*a*_*i*_,*a*_*j*_}, which are the alleles of the shared parent, such that each individual possesses at least one copy of either *a*_*i*_ or *a*_*j*_ at each respective locus; this describes the requirement that the half-sibling group inherits from the common parent.

## Forced allele incompatibilities

When populations are completely monogamous, determining whether each family in a population structure has valid parent genotypes is trivial, as decisions made in reconstructing the parents of one sibgroup are independent of all other families. However, when reconstructing half-sibling populations, determining whether there is a set of parents and matings that can explain a collection of identified sibgroups under Mendelian inheritance assumptions is much more difficult. For any individual, choosing its father’s genotype uniquely determines which allele must have been inherited from, and hence present in, the mother, unless the offspring genotype exactly matches the paternal one. Thus, the decision affects the maternal genotype. In polygamous populations, this influence on the choice of maternal genotype by paternal genotype also indirectly affects the choice of genotype for each other father that the common mother has mated with. For example, if both maternal alleles at a locus are fixed by one sibgroup’s reconstruction, then any other alleles found in offspring from a different mating of the same mother must have been inherited from the father.

For highly polygamous populations with many indirect inheritance relationships of this sort, it can be difficult to determine whether a proposed population structure is valid. We show that deciding whether there is a valid parental genotype for each half-sibling partition in a candidate half-sibling population reconstruction is *NP*-Complete. Thus, we cannot expect polynomial time algorithms to produce valid parental genotypes, even when they exist. We instead propose an integer program, which identifies the minimum number of individuals that must be removed from a candidate population reconstruction so that the resulting population is valid under Mendelian inheritance, and give experimental results of using it.

### Complexity of the valid half-sibling partitioning decision problem

Given maternal and paternal half-sibling partitionings, with each individual belonging to exactly one maternal and one paternal partition, is it possible to assign genotypes to the parents of each half-sibling family in a way that respects the property that every individual must inherit one of exactly two alleles from each parent at each locus? We will call this problem VALID HALF-SIBLING PARTITIONING.

**Theorem 1. ***VALID HALF-SIBLING PARTITIONING is NP-complete*.

*Proof.* We first show that VALID HALF-SIBLING PARTITIONING ∈ *NP*. Given an instance of the problem and an assignment of genotypes to the parents of each half-sibling family, we can verify in polynomial time whether or not the population structure is valid. The algorithm is straight-forward: for each heterozygotic child, check that the allele inherited from the mother is not the same as the allele inherited from the father. When the parent and child genotypes differ, we say that the child is forced to inherit a specific allele, *e.g.* child (*a*,*b*) is forced to inherit allele *a* from mother (*a*,*c*). If the parent does not force an allele because the parent genotype is identical to the child’s genotype, then that child cannot cause an incompatibility: the inherited allele from the identical parent is whichever allele was not inherited from the child’s other parent.

Next, we give a polynomial-time reduction from the *NP*-complete MONOTONE ONE-IN-THREE SAT problem to VALID HALF-SIBLING PARTITIONING. The MONOTONE ONE-IN-THREE SAT problem is: given a set of boolean clauses, each containing three non-negated literals, determine whether a configuration of literals exists such that exactly one literal in each clause is set true. This problem is also called EXACT-COVER-BY-3-SETS (X3C), which was used in the first proof of the NP-hardness of parsimony phylogeny [15]. The reduction requires three gadgets that translate literals and clauses in a MONOTONE ONE-IN-THREE-SAT instance into alleles and families in a VALID HALF-SIBLING PARTITIONING instance, respectively.

The first gadget translates picking a literal in a clause to picking a parent for a family. The second gadget defines paternal families that introduce additional necessary offspring. From the MONOTONE ONE-IN-THREE SAT perspective, the third gadget enforces the rule that if a literal is chosen to be set true in one clause, it must be chosen to be true in all of the clauses it belongs to.

We begin by defining a one-to-one function *f* : *x*→*y* which assigns each SAT literal to a unique integer allele value. Assume also that there are *m* clauses.

1. The *selection gadget* creates a maternal family with three possible mothers and six offspring for each clause in the SAT instance by mapping literals in a clause to alleles in a family. For the clause (*x*_*i*_∨*x*_*j*_∨*x*_*k*_), the corresponding *y*_*i*_, *y*_*j*_, and *y*_*k*_ will be the alleles present in the created family. Six children are created by making two copies of each pairwise grouping of the *y* alleles: for this clause, we would create the maternal groups in Table 1.

**Table 1:** **Selection gadget for clause (*x***_***i***_**∨** ***x***_***j***_**∨** ***x***_***k***_**)**

		**Genotype of**
**Literals**	**Family**	**possible shared parent**
*x*_*i*_	(*y*_*j*_,*y*_*k*_)_0_	(*y*_*j*_,*y*_*k*_)
	(*y*_*j*_,*y*_*k*_)_1_	
*x*_*j*_	(*y*_*i*_,*y*_*k*_)_0_	(*y*_*i*_,*y*_*k*_)
	(*y*_*i*_,*y*_*k*_)_1_	
*x*_*k*_	(*y*_*i*_,*y*_*j*_)_0_	(*y*_*i*_,*y*_*j*_)
	(*y*_*i*_,*y*_*j*_)_1_	

In this half-sibgroup, there are three choices for the maternal genotype: (*y*_*i*_,*y*_*j*_), (*y*_*i*_,*y*_*k*_), and (*y*_*j*_,*y*_*k*_). Choosing, for example, maternal genotype (*y*_*j*_,*y*_*k*_) corresponds to setting literal *x*_*i*_ to true in the MONOTONE ONE-IN-THREE SAT instance: the rule is that the allele not present in the maternal genotype is the true literal. There are total of *m* of these maternal families, each with six members.

2. The *mapping gadget* creates two paternal families for each potential mother, for a total of six paternal families per maternal selection gadget family. The gadget highlights which literal is set to true in the clause corresponding to the selection gadget.

The 6*m* mapping families introduce new alleles *s*_0_…*s*_*m*−1_, one for each clause, and another distinct allele *z* common to all of the mapping families. The *s*_*i*_ alleles are used in the third gadget to enforce that, once a literal is set to true in one clause, it is true in every clause.

We now show how to construct the paternal families using the (*y*_*i*_,*y*_*j*_) children from the selection gadget. Let *k*_*i*_ be the number of clauses that contain variable *x*_*i*_. Each paternal family *p* containing the *y*_*i*_ allele must have ki2 copies of the offspring (*s*_*p*_,*y*_*i*_)_0_ and (*s*_*p*_,*y*_*i*_)_0_. For the clause (*x*_*i*_∨*x*_*j*_∨*x*_*k*_), we create the six families in Table 2 (though here we only show one copy of (*s*_*p*_,*y*_*i*_)_0_ and (*s*_*p*_,*y*_*i*_)_1_.

**Table 2:** **Paternal families for clause (*x***_***i***_**∨** ***x***_***j***_**∨** ***x***_***k***_**)**

***y***_***i***_** fam.**	***y***_***i***_** fam.**	***y***_***j***_** fam.**	***y***_***j***_** fam.**	***y***_***k***_** fam.**	***y***_***k***_** fam.**
(*y*_*i*_,*y*_*j*_)_0_	(*y*_*i*_,*y*_*k*_)_0_	(*y*_*i*_,*y*_*j*_)_1_	(*y*_*j*_,*y*_*k*_)_0_	(*y*_*i*_,*y*_*k*_)_1_	(*y*_*j*_,*y*_*k*_)_1_
(*s*_*p*_,*y*_*i*_)_0_	(*s*_*p*_,*y*_*i*_)_1_	(*s*_*p*_,*y*_*j*_)_0_	(*s*_*p*_,*y*_*j*_)_1_	(*s*_*p*_,*y*_*k*_)_0_	(*s*_*p*_,*y*_*k*_)_1_
(*s*_*p*_,*z*)_0_	(*s*_*p*_,*z*)_1_	(*s*_*p*_,*z*)_2_	(*s*_*p*_,*z*)_3_	(*s*_*p*_,*z*)_4_	(*s*_*p*_,*z*)_5_
(*y*_*j*_,*z*)_0_	(*y*_*k*_,*z*)_0_	(*y*_*i*_,*z*)_0_	(*y*_*k*_,*z*)_1_	(*y*_*i*_,*z*)_1_	(*y*_*j*_,*z*)_1_

Consider the set of six mapping gadget families with alleles {*y*_*i*_,*y*_*j*_,*y*_*k*_}, corresponding to the clause *c*_*p*_ = (*x*_*i*_∨*x*_*j*_∨*x*_*k*_). If, for example, the *y*_*i*_ allele for all copies of offspring (*s*_*p*_,*y*_*i*_)_0_ and of (*s*_*p*_,*y*_*i*_)_1_ is inherited from its father, then the corresponding maternal selection parent must be (*y*_*j*_,*y*_*k*_), indicating that variable *x*_*i*_ is set to true. Again without loss of generality, if the *s*_*p*_ allele is inherited from the father, then the maternal parent from the selection gadget cannot possibly be (*y*_*j*_,*y*_*k*_).

3. Lastly, we construct a gadget that maintains consistency in each use of the variables, from the SAT instance perspective. That is, it forces each true literal to be true and each false literal to be false in every clause in which that literal appears. The *enforcement gadget* constructs a constraining maternal family for each pair of clauses in which a common literal occurs. For instance, if literal *x*_*i*_ appears in clauses *c*_*p*_ and *c*_*q*_, then a *c*_*p*_/*c*_*q*_ enforcment family will be constructed so that either *y*_*i*_ is forced to be maternal or *s*_*p*_ and *s*_*q*_ are forced to be maternal. The gadget makes use of the (*s*_*p*_,*y*_*i*_)_∗_ and (*s*_*q*_,*y*_*i*_)_∗_ individuals created by the mapping gadget. The families created for a literal *x*_*i*_ that appears in clauses *c*_*p*_, *c*_*q*_, and *c*_*r*_ are found in Table 3.

**Table 3:** **Enforcement gadget for variable** ***x***_***i***_**, appearing in** ***c***_***p***_**,** ***c***_***q***_** and** ***c***_***r***_

***c***_***p***_**/*c***_***q***_	***c***_***p***_**/*c***_***r***_	***c***_***q***_**/*c***_***r***_
(*s*_*p*_,*y*_*i*_)_0_	(*s*_*p*_,*y*_*i*_)_0_	(*s*_*q*_,*y*_*i*_)_0_
(*s*_*p*_,*y*_*i*_)_1_	(*s*_*p*_,*y*_*i*_)_1_	(*s*_*q*_,*y*_*i*_)_1_
(*s*_*q*_,*y*_*i*_)_0_	(*s*_*r*_,*y*_*i*_)_0_	(*s*_*r*_,*y*_*i*_)_0_
(*s*_*q*_,*y*_*i*_)_1_	(*s*_*r*_,*y*_*i*_)_1_	(*s*_*r*_,*y*_*i*_)_1_
(*s*_*p*_,*z*)_0_	(*s*_*p*_,*z*)_1_	(*s*_*q*_,*z*)_1_
(*s*_*q*_,*z*)_0_	(*s*_*r*_,*z*)_0_	(*s*_*r*_,*z*)_1_

Each enforcement gadget family for *y*_*i*_ has two copies of (*s*_*p*_,*y*_*i*_) which are the two children from the mapping gadgets containing (*y*_*i*_,*y*_*j*_) and (*y*_*i*_,*y*_*k*_). If *s*_*p*_ is forced, then *s*_*q*_ must also be forced to avoid an incompatibility. As a result, *y*_*i*_ is forced in both paternal mapping gadget families, indicating that *x*_*i*_ is kept true in both cases. As stated in the mapping gadget, this forces which maternal parent must be chosen in the selection gadget. However, if *y*_*i*_ is forced in the enforcement gadget, then *s*_*p*_ and *s*_*q*_ are forced to be true in the paternal mapping gadget families, which excludes the parents corresponding to *x*_*i*_ in the SAT instance from being chosen in the respective selection gadget families.

Finally, for all individuals with only a single assigned parent, which is true for all individuals with the allele *z*, we assign them to a single-element family corresponding to their unassigned parent, thus enforcing no further restrictions on parental genotypes.

In the MONOTONE ONE-IN-THREE SAT problem, a literal from each clause must be set to true. The selection gadget translates the task of choosing an allele to picking the parents of maternal families. Each selection gadget family contains three distinct alleles {*y*_*i*_,*y*_*j*_,*y*_*k*_}. Choosing maternal parent (*y*_*i*_,*y*_*j*_) is equivalent to setting *x*_*k*_ true and the *x*_*i*_ and *x*_*j*_ literals to false.

Finally, let *n* be the number of literals and *m* be the number of clauses. Constructing the VALID HALF-SIBLING PARTITIONING instance requires *O*(*m*) children for the first gadget, *O*(*m*^2^·*n*) additional children for the second gadget, and *O*(*m*^2^) additional children for the third gadget. Since *m* ≤ *n*, the resulting transformation is polynomial in the number of literals. □

As an example of this reduction, consider the MONOTONE ONE-IN-THREE SAT instance (*x*_1_ ∨ *x*_2_ ∨ *x*_3_) ∧ (*x*_1_ ∨ *x*_4_ ∨ *x*_5_). Its maternal selection and enforcement gadget families are in Table 4 and its paternal mapping gadget families are in Table 5.

**Table 4:** Maternal selection and enforcement gadgets for example SAT instance

***M***_**0**_	***M***_**1**_	***M***_**2**_
(**1**,2)_0_	(**1**,4)_0_	(*s*_0_,**1**)_0_
(**1**,2)_1_	(**1**,4)_1_	(*s*_0_,**1**)_1_
(**1**,3)_0_	(**1**,5)_0_	(*s*_0_,**1**)_1_
(1,**3**)_1_	(1,**5**)_1_	(*s*_1_,**1**)_1_
(2,**3**)_0_	(4,**5**)_0_	(*s*_0_,**z**)_6_
(2,**3**)_1_	(4,**5**)_1_	(*s*_1_,**z**)_6_

**Table 5:** Paternal mapping gadget families for example SAT instance

***P***_**0**_	***P***_**1**_	***P***_**2**_	***P***_**3**_	***P***_**4**_	***P***_**5**_
(1,**2**)_0_	(1,**3**)_0_	(**2**,3)_0_	(1,**2**)_1_	(**1**,3)_1_	(**2**,3)_1_
(*s*_0_,1)_0_	(*s*_0_,1)_1_	(*s*_0_,**2**)_0_	(*s*_0_,**2**)_1_	(*s*_0_,3)_0_	(*s*_0_,3)_1_
(*s*_0_,*z*)_0_	(*s*_0_,*z*)_1_	(*s*_0_,**z**)_2_	(*s*_0_,**z**)_3_	(*s*_0_,*z*)_4_	(*s*_0_,*z*)_5_
(**2**,*z*)_0_	(**3**,*z*)_0_	(3,**z**)_1_	(1,**z**)_0_	(**1**,*z*)_1_	(**2**,*z*)_1_
***P***_***6***_	***P***_***7***_	***P***_***8***_	***P***_***9***_	***P***_***10***_	***P***_***11***_
(1,**4**)_0_	(1,**5**)_0_	(**4**,5)_0_	(1,**4**)_1_	(**1**,5)_1_	(**4**,5)_1_
(*s*_1_,1)_0_	(*s*_1_,1)_1_	(*s*_1_,**4**)_0_	(*s*_1_,**4**)_1_	(*s*_1_,5)_1_	(*s*_1_,5)_1_
(*s*_1_,*z*)_0_	(*s*_1_,*z*)_1_	(*s*_1_,**z**)_2_	(*s*_1_,**z**)_3_	(*s*_1_,*z*)_4_	(*s*_1_,*z*)_5_
(**4**,*z*)_0_	(**5**,*z*)_0_	(5,**z**)_1_	(1,**z**)_2_	(**1**,*z*)_3_	(**4**,*z*)_1_

There are several feasible solutions to the M-1-3-SAT instance, but the example illustrates the case where literals *x*_2_ and *x*_4_ are set true in the M-1-3-SAT instance. The inherited allele for each individual in each family is bolded to represent the corresponding VHSP solution where mothers (1,3) and (1,5) are chosen in the selection gadget.

### Identifying allele incompatibilities

Unfortunately, the *NP*-completeness of the VHSP problem makes it very unlikely that a polynomial time algorithm exists for verifying that given maternal and paternal partitions have valid parental genotype assignments. Therefore, we present a 0/1 integer program that identifies individuals to remove to obey Mendelian inheritance. We use parsimony and select the fewest individuals to remove.

We now present a 0/1 integer program to solve this problem.

$$\begin{array}{lll} & \;\text{minimize}\;\underset{i}{\sum }x_{i}\; & \;\\ & \text{subject to}\; & \;\\ & \;\;\;\;\;\;\begin{array}{ll} \underset{k\in K}{\sum }y_{j,k}^{l}\leq 2,\; & 0\leq j<|C|,0\leq l<m\\ x_{0,i}^{l}+\frac{1}{2}(y_{\pi_{0}(i),\lambda_{0}(i)}^{l}+y_{\pi_{1}(i),\lambda_{1}(i)}^{l})\geq 1,\; & \;0\leq i<n,0\leq l<m\\ x_{1,i}^{l}+\frac{1}{2}(y_{\pi_{0}(i),\lambda_{1}(i)}^{l}+y_{\pi_{1}(i),\lambda_{0}(i)}^{l})\geq 1,\; & \;0\leq i<n,0\leq l<m\\ x_{0,i}^{l}+x_{1,i}^{l}-x_{i}^{l}\leq 1,\; & \;0\leq i<n,0\leq l<m\\ x_{i}-x_{i}^{l}\geq 0,\; & \;0\leq i<n,0\leq l<m \end{array}\; & \; \end{array}$$

For a population with *n* individuals, genotyped at *m* loci, let *x*_*i*_ = 1 denote the decision to remove individual *i* from the population. The variable $y_{j,k}^{l}$ represents the binary choice of having the *k*^*th*^ allele in the common parent of the family *j* at locus *l*. Denote the multi-set which contains the maternal and paternal families as C, and let *π*_0_ and *π*_1_ be functions that map an individual to its maternal and paternal index in C respectively. Finally, let *λ*_0_ and *λ*_1_ map the first and second allele of an individual to an index in *K*, the set of all alleles.

The first constraint enforces that no parent can have more than two alleles. The second and third constraints enforce Mendelian mating requirements on individuals: an individual is invalid, and hence must be removed if it does not receive one allele from its mother and its other allele from its father. There are two possible ways to satisfy this constraint. Either the child received its first listed allele from its mother and the second allele from its father or vice versa. The two constraints are the logical or of these two possibilities, and the fourth constraint identifies incompatible loci as those that are unsatisfied by either of these possibilities. Finally, the last constraint enforces the requirement that *x*_*i*_ must be 1, corresponding to individual *i* being selected for removal, if the individual has any incompatible loci.

We note that the minimization objective forces *x*_*i*_ and $x_{i}^{l}$ to be 0 as often as possible. Since there can never be more families than individuals, the integer program has a total of *O*(*m*·*n*) constraints.

## Clustering half-sibling families

Sibship reconstruction finds a population clustering which obeys Mendelian genetics. In the full sibling clustering F, each individual appears only once. For half-siblings, an algorithm must construct ℳ and P when both sexes are polygamous or only one of the two partitions when one sex is monogamous. Here, we describe the approach of SibJoin, our program for identifying half-sibling populations.

### Measuring similarity

For a given half-sibling input, SibJoin relies on an *n*×*n* similarity matrix which expresses the similarity between each pair of individuals in the offspring population set. Brown and Berger-Wolf [13] used a similarity measure which, for each pair of individuals, is the count of third individuals in the population that form a compatible full sibling triplet with the pair. They proved that any incompatible candidate full sibling group must contain an incompatible triplet and give a probabilistic argument that pairs of individuals with large numbers of compatible triplets are likely siblings. Unfortunately, the half-sibling property is much weaker because it only operates on one parent. Ruling out a potential half-sibling group can take as many as six individuals, compared to the three that is required for full siblings.

**Theorem 2. ***There exist incompatible half-sibling groups for which their smallest incompatible subgroup has six members*

*Proof.* Consider the sextet of individuals with one locus and four alleles {[ (1,2)],[ (1,3)],[ (1,4)],[ (2,3)], [ (2,4)],[ (3,4)]}. Any five of the individuals form a valid half-sibship under the half-sibling property, but the incompatibility appears when all six individuals are examined together: the common half-sibling parent would need three alleles at the locus. □

The lower bound suggests that examining triplets for half-siblings could yield a falsely high count when individuals are not actually related. Additionally, the probability that three random individuals form a valid half-sibship is much higher than that of three individuals forming a valid full sibship. By enumerating all possible triplets with a pool of five alleles, we see that 96.62*%* of all triplets are compatible under the half-sibling property, but only 56.61*%* of all triplets are compatible under the full sibling properties: that is, incompatibilities are not nearly so much of a warning of unrelatedness for half-sibling reconstruction as for full-sibling reconstruction. If the number of alleles is set to ten, then 75.46*%* of half-sibling triplets are compatible (most often, these result when any two individuals in the triple share an allele), while the number of full sibling compatible triplets is just 20.94*%*.

In the place of a triplet-based similarity function, SibJoin uses a pairwise measure. Given two individuals, each with *m* loci, we count shared alleles at each locus independently, between the two individuals. For example, the pair of individuals *x* = [ (1,2),(2,2),(1,3)] and *y* =[ (1,1),(2,2),(2,3)] has similarity *s*_*xy*_ = 4. The pairwise similarity matrix for this simple measure may be computed in *O*(*n*^2^*m*) time, as opposed to the *O*(*n*^3^*m*) time that is required by the brute-force triplet method.

Let *X* be the random variable that represents the number of shared alleles between two individuals at a single locus. If we assume an even allele distribution, then the expected number of shared alleles at a single locus is given in Eq. 1, 2, and 3.

(1) $$\begin{array}{ll} \text{E}[\;X|\text{full siblings}] & ={\left(\frac{k-1}{k}\right)}^{2}\cdot \frac{4k^{2}+3k-2}{4k^{2}}+\frac{3k-1}{k^{2}}\; \end{array}$$

(2) $$\begin{array}{ll} \text{E}[\;X|\text{half siblings}] & =\frac{k-1}{k}\cdot \frac{k^{2}+3k-1}{2k^{2}}+\frac{1}{k}\left(1+\frac{1}{k}\right)\; \end{array}$$

(3) $$\begin{array}{ll} \text{E}[\;X|\text{unrelated}] & =\frac{4k^{2}-4k+2}{k^{3}}\; \end{array}$$

Assuming the parents of two full siblings are each heterozygotic, two siblings have a 50% chance of inheriting the same allele from each parent: if either parent is homozygotic, then the offspring are guaranteed to inherit the same allele from that parent. Similarly, half-siblings have a 50% chance of inheriting the same allele from their heterozygotic common parent. For unrelated individuals, the expected number of shared alleles approaches 0 as the number of distinct alleles at a locus grows. When there are many possible alleles, it is unlikely that two unrelated individuals will inherit the same alleles. So, as *k* grows without bound **E**[ *X*|full siblings]→1, $\text{E}[\;X|\text{half siblings}]\to \frac{1}{2}$, and **E**[ *X*unrelated]→0.

Additionally, we may apply Hoeffding’s inequality to show that the probability that a pairwise allele similarity deviates far from its mean decreases exponentially as the number of loci increases. Let *X* be a random variable as described above. For independent loci, the allele similarity *x*_*i*_ is the allele similarity of the *i*’th locus with 0 ≤ *X*_*i*_ ≤ 2 for 1 ≤ *i* ≤ *m*. By application of Hoeffding’s inequality to the mean allele similarity $\bar{X}=\overset{m}{\underset{i=0}{\sum }}X_{i}/m$,

(4) $$\text{Pr}(|\bar{X}-\text{E}[\;\bar{X}]|\geq t)\leq 2\cdot \text{exp}\left(-\frac{t^{2}m}{2}\right)$$

Therefore, the pairwise similarity measure will perform well when either the number of alleles or loci is sufficiently large: it easily separates unrelated individuals from half siblings and full siblings. Our results also indicate that pairwise similarity method performs well, even with modest numbers of alleles and loci.

### Joining individuals

**Figure 1:**
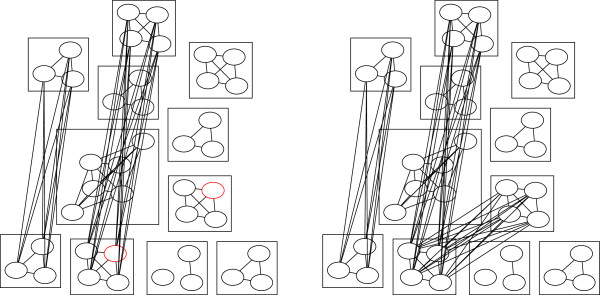
**Demonstration of a successful iteration of SibJoin.** Nodes represent individuals, edges represent a half or full sibling relationship constructed by the algorithm, and nodes which share a box represent true full siblings.

SibJoin’s half-sibling clustering uses the observation that individuals with high allele similarity are very likely half or full siblings. SibJoin begins with 2*n* clusters, each of which contains a single individual. Every individual appears in exactly two clusters, representing its maternal and paternal half-sib groups. SibJoin uses a variant of single linkage clustering to join clusters. Single linkage clustering is a form of agglomerative clustering that determines the similarity of two clusters *C*_*i*_ and *C*_*j*_ by computing *sim*(*C*_*i*_,*C*_*j*_) = max*x*∈*C*_*i*_,*y*∈*C*_*j*_*s*_*xy*_, and then joins the two compatible groups with highest similarity. A sample join is demonstrated in Figure 1. Ties in similarity are broken by joining the groups with the highest combined number of members first since large compatible half-sibling groups are more likely to be related than small groups. SibJoin’s success comes from two observations. First, in order for bad joins to occur between any pair of individuals *i* and *j*, the similarity between *i* and *j* would need to be larger than the similarity between *i* and each of *i*’s real half-siblings, and likewise for *j*. Secondly, as clusters grow, the odds that two unrelated clusters form a compatible half-sibship rapidly diminishes.

Joining must only occur if two clusters form a valid half-sibship. At the initialization of the algorithm, each individual is assigned a feasible parent set with size at most *O*(*k*) per locus. Each join results in a parent set which is the intersection of the parent sets from the two clusters. If the intersection produces the null set, then there is no parent which can explain the new cluster and the join is rejected. Therefore, testing whether or not a join is valid takes *O*(*km*) time. When a site experiences allelic dropout, SibJoin makes no assumptions about its parental restrictions; however, sites with (∗,∗) are never counted toward allele similarity between individuals.

Unlike crisp clustering methods which mandate that each individual appear in exactly one cluster, the half-sibling problem contains both a maternal and paternal group for each individual. We enforce the restriction that any set of individuals sharing both a maternal and paternal cluster must be compatible full siblings under the 4-allele and 2-allele properties by maintaining a clustering of full siblings. Because incompatible full sibling groups are less likely than incompatible half-sibling groups of the same size, at each similarity step SibJoin joins clusters which form valid full sibships first.

Microsatellites give no information about which alleles are maternal and which are paternal. Since SibJoin constructs families in an iterative manner, part of a maternal family could be reconstructed in the maternal partitioning, while the other part of the family is constructed in the paternal partitioning. If we are strict about which sets we call maternal and paternal, then the two halves will never be joined and the half on the paternal side will likely force incorrect future joins. Our solution is to implement a bipartite graph *G* = (*V*,*E*) where each cluster is a vertex and edges exist between clusters which share an individual. Let a join between clusters *C*_*i*_ and *C*_*j*_ be an event which combines *C*_*j*_ into *C*_*i*_ and let *E*(*v*) be the set of edges that touch *v*. In our graph, *join*(*C*_*i*_,*C*_*j*_) results in $E(v_{i}):=E(v_{i})⋃E(v_{j})$ followed by the removal of *v*_*j*_ and all edges in *E*(*v*_*j*_). Enforcing bipartiteness as a post-condition of the join operation allows flexibility while ensuring that the solution results in each individual having one parent of each sex.

## Results and discussion

We evaluated SibJoin’s performance with simulated and real population data. The experimental results from real populations are contrasted with the the HS-MSC and COLONY half-sibling approaches.

### Accuracy measure

Partition distance is a metric which measures the distance between two partitions as the minimum number of individuals that must be removed from a population until the two clusterings are identical. This metric is widely used in sibship reconstruction literature and in bioinformatics in general [16,17]. When true partitionings are known, partition distance may be used to compute the true accuracy of an algorithm; however, it may also be used to assess changes between candidate sibships for which ground truth is not known [18].

Despite its prominence in the kinship analysis literature, partition distance offers only a coarse estimate of correctness because it disregards how the excluded individuals are constructed within the partitioning. For example, failing to join two related partial families is less destructive than incorrectly joining one of the partial families to an unrelated family. However, in both instances, the partition distance is identical. A concrete example is given in Meilă [19].

Instead, we use an information-theoretic metric called variation of information (VI) [19]. The VI measures how much the information given by two clusters differ and is preferable because it quantifies the amount of uncertainty introduced by misplaced individuals.

The VI between two partitionings is 0 if and only if the two partitionings are identical. Smaller VI corresponds to more similar partitionings. Like entropy, the VI is always non-negative. It also has a tight upper bound of log*n*[19]; therefore, we normalize VI to a value in [0,1] before reporting the score for each of our trials.

For half-siblings, calculating the VI is not straight-forward because we have two partitionings, maternal and paternal, instead of the single partitioning that is common in most clustering problems. Since there are two clusterings, we compute the average variation of information between the two maternal clusters, ℳ and ${ℳ}^{\prime }$, and the two paternal clusters P and $P^{\prime }$, where ℳ and P are the true partitionings. Since it is usually impossible to determine the sex of the common parent, we calculate two different VI values and choose the sex assignment that minimize the VI.

(5) $$HS_{\text{VI}}=\frac{\min\left(\frac{\text{VI}(ℳ,{ℳ}^{\prime })+\text{VI}(P,P^{\prime })}{2},\frac{\text{VI}(ℳ,P^{\prime })+\text{VI}(P,{ℳ}^{\prime })}{2}\right)}{\log n}$$

### Simulated data set results

We constructed simulation sets to test various parameters. Our model generates individuals from an equal number of mothers and fathers. Parents are chosen randomly, and children are generated from mother-father pairs according to an even allele distribution. Simulated data had default parameter values of 6 alleles, 6 loci, half-sibling family sizes of 5 individuals, and a population size of 40 individuals. The results are an average of ten trials per parameter value. Trials which failed to complete in 1 day are reported as ’-’. The population size was increased to 80 individuals for family size trials so that the partitionings did not become trivial. The loci count was increased to 10 and family size to 20 when testing population sizes above 200 individuals. A summary of our parameter tests and their results may be found in Table 6. Testing occurred on a 2.66 GHz machine, containing 8 GB of RAM, and running Python 2.7.

**Table 6:** Simulated test results for SibJoin and COLONY 2.0 averaged over 10 trials

**Fixed**	**Parameter**		SibJoin		COLONY 2.0
**parameter**	**settings**	**Runtime**	**VI (normalized)**	**Runtime**	**VI (normalized)**
	2	2.8 ms	0.396	48.9 min	0.553
	5	13.2 ms	0.222	19.7 min	0.110
*k*: number of alleles	10	6.7 ms	0.014	12.8 min	0.004
	15	5.1 ms	0.014	10.2 min	0.006
	20	5.7 ms	0.003	10.0 min	0.000
	2	8.7 ms	0.469	10.7 min	0.524
	5	10.1 ms	0.156	17.2 min	0.130
*m*: number of loci	10	11.1 ms	0.035	14.2 min	0.001
	15	12.7 ms	0.002	20.4 min	0.000
	20	12.1 ms	0.000	21.3 min	0.000
	10	0.4 ms	0.042	2.29 min	0.343
*n*: population size	50	16.8 ms	0.104	17.1 min	0.078
	100	82.5 ms	0.201	73.5 min	0.086
	200	3.31 sec	0.230	-	-
	500	34.68 sec	0.013	-	-
	1000	2.84 min	0.015	-	-
	2000	12.43 min	0.018	-	-
	1	51.9 ms	0.546	-	-
*f*: family size	5	51.1 ms	0.183	29.6 min	0.051
	10	46.2 ms	0.040	19.6 min	0.017
	20	58.4 ms	0.009	21.7 min	0.042

Trials which did not complete in 24 hours are marked ‘-’.

In most cases, the reported VI score approximates the ratio of partition distance to population size. Overall, COLONY 2.0 was more accurate, but took thousands of times longer, often with only small gains in accuracy. SibJoin does much worse than COLONY 2.0 on the 10 allele test set, but the discrepancy is due to a single trial for which SibJoin receives a VI of 0.084 while COLONY 2.0 produces a perfect reconstruction. For the 10 loci test set, SibJoin’s VI is again higher, but in practice the false positive difference between it and COLONY 2.0 is about one individual per trial.

SibJoin does worst when the population size is large and the family size is small. For instance, when tested with a 100 individual population and families of 5 individuals, SibJoin rendered a VI of 0.201 compared to COLONY 2.0’s VI of 0.086. When family sizes are small and population sizes are large, it is much more likely for two unrelated individuals to be mistakenly labeled as half-siblings due to the explanations given in section 4.3. However, SibJoin’s accuracy rapidly improves with modest increases in family size. In fact, SibJoin is more accurate than COLONY 2.0 in trials with families containing 20 individuals. Unsurprisingly, both methods poorly reconstruct populations where only two alleles are present. With only two alleles, all individuals can be full or half-siblings.

We may also use SibJoin to explore populations with extreme numbers of individuals. SibJoin was able to reconstruct populations of 500, 1000, and 2000 individuals in under 10 minutes, yet problems of this magnitude are intractable for the HS-MSC and both of the COLONY programs.

### Biological data set results

SibJoin was tested on two biological data sets. The first data set is a population of 112 field crickets with 7 mothers and 6 sampled loci [20]. The second data set is a population of 672 kelp rockfish with 7 mothers and 7 sampled loci [21]. Results are shown in Table 7. Neither COLONY 2.0 nor the HS-MSC produced a solution for the 672 rockfish population, so samples from three of the parents were taken to reduce the population size to 288 individuals. In both populations, only maternal parentage was available. For all trials, SibJoin was run in a configuration that only attempts to reconstruct the maternal sex.

**Table 7:** Tests for biological data

**Data set**	**Algorithm**	**Runtime**	**VI (normalized)**	**False positives**
112 crickets	COLONY 2.0	35.7 min	0.000	0
	HS-MSC	-	n/a (see caption)	2
	SibJoin	19.3 ms	0.014	1
288 kelp rockfish	COLONY 2.0	624.5 min	0.000	0
	HS-MSC	-	n/a (see caption)	0
	SibJoin	87.5 ms	0.000	0
672 kelp rockfish	COLONY 2.0	-	-	-
	HS-MSC	-	-	-
	SibJoin	5.02 sec	0.108	78

A ‘-’ indicates that an algorithm did not complete after 24 hours. SibJoin was the only algorithm able to construct a solution for a 672 individual population of rockfish. The variation of information is not computed for the HS-MSC since it allows instances of the same individual, which causes ill-defined VI scores.

Our results are compared to the HS-MSC results in [7] and to our own benchmarks on COLONY 2.0. Because the HS-MSC is not publicly available, we could not assess runtime information for the program. However, the authors do note that the HS-MSC IP finished in under one day. The difference between the two runtimes is not explained merely by CPU speed increases across a small number of years. Additionally, neither COLONY 2.0 nor the HS-MSC’s half-sibling minimum set cover approach constructed a feasible answer for the 672 rockfish data set: COLONY 2.0 was stopped after running for three days. SibJoin constructs an accurate solution in under 10 seconds.

The HS-MSC ILP does not enforce that individuals must have one parent of each sex and both partition distance and variation of information are ill-defined when the result is not a true partitioning. In the population of 112 crickets, the HS-MSC had two false positives and was otherwise correct. In the test set containing 288 rockfish, HS-MSC had 4 false positives and was otherwise correct. COLONY 2.0 was correct in all instances. SibJoin correctly reconstructed the half-sibship for the 288 rockfish and only misplaced one individual in the cricket test. SibJoin was the only algorithm to complete for the population of 672 rockfish. Overall, SibJoin is as accurate as the HS-MSC and nearly as accurate as COLONY 2.0, but is much faster than either: SibJoin solves the small rockfish instance over 42,000 times faster than COLONY 2.0.

### Minimum population removal IP results

Using the integer program outlined previously, we can identify the minimum-size set of individuals which must be removed in order to make a SibJoin solution feasible. We assume that this set is small, so finding the minimum individuals to remove should capture many incorrect individuals.

Although the IP generally solves quickly, it struggles to find a global optimum for populations of hundreds of individuals. In these cases the IP gets very close, often with integrality gaps below 3%, but never reaches an optimal integer solution since it runs out of memory. In our experiments, we enforce a 5 minute time limit on the IP. We report the percent of trials that failed to reach integrality in Table 8. An approximate solution is acceptable as long as there is a reliable way to correctly re-add identified individuals into the population; also, of course, there is no reason to assume that the smallest set to remove consists of those that are causing trouble.

**Table 8:** SibJoin trials with forbidden allele detection

**Fixed parameter**	**Parameter**	**Norm. VI**	**FP**	**Recall**	**Precision**	**Timeout rate**
*k*: number of alleles	2	0.396	25.9	0.000	0.000	0.0
	5	0.225	12.3	0.300	0.694	0.0
	10	0.013	0.2	0.000	0.000	0.0
	15	0.014	0.0	-	-	0.0
	20	0.003	0.0	-	-	0.0
*m*: number of loci	2	0.491	23.7	0.109	0.563	0.0
	5	0.150	6.6	0.355	0.537	0.0
	10	0.032	1.2	0.62	0.650	0.0
	15	0.002	0.0	-	-	0.0
	20	0.000	0.0	-	-	0.0
*n*: population size	10	0.042	0.5	0.2	1	0.0
	50	0.098	10.2	0.340	0.679	0.0
	100	0.201	41.0	0.400	0.765	0.1
	200	0.220	88.9	0.408	0.780	1.0
*f*: family size	1	0.527	58.5	0.317	0.778	0.7
	5	0.181	22.8	0.439	0.756	0.0
	10	0.038	3.6	0.313	0.477	0.0
	20	0.009	1.4	0.000	0.000	0.0

A ‘-’ occurs when there are no false positives.

Table 8 reports the recall and precision of the IP: the percentage of all incorrect individuals that are identified by the IP and the percentage of individuals that are actually incorrect among the individuals identified by the IP. We find that the integer program can have a poor recall, finding only 30% of the false positives in some situations; however, the precision is relatively high. For individuals in the minimum removal set, the number of incorrectly placed individuals is consistently above 50%. The precision is significant since SibJoin’s total error rate is often far below 50%. If we found a way to correctly reintroduce the set of individuals identified by the IP, then the overall SibJoin error rate would decrease significantly.

The IP does worst when there are only two alleles or two loci. This is unsurprising since there will be no incompatibilities when each locus contains less than three alleles and data with few loci have smaller risk of forbidden allele structures with bad joins. However, both recall and precision tend to increase with population size as demonstrated by the 100 and 200 population size test cases. For populations with 200 individuals, the IP did not reach integrality within 5 minutes, but still produced solutions with high recall and precision relative to the other tests, indicating that the IP is still useful at identifying mis-placed individuals in large populations.

## Conclusions

It is important to be able to determine whether or not a proposed population structure is valid under Mendelian inheritance assumptions. For half-siblings, we have proved that even determining if such a structure obeys Mendelian laws is *NP*-complete, which is surprising since the same determination in monogomous populations is trivial. This result has important implications for half-sibling algorithms in general since most existing algorithms do not specifically enforce which allele is inherited from which parent and those that do very likely have running times which are exponential in the size of the population. We have also provided an integer program that solves an optimization variant of the problem: what is the minimum number of individuals that must be removed from a population in order for the population structure to be valid. The IP was run against SibJoin’s population reconstructions. Although the IP only had a recall of 30 to 40 percent when run against SibJoin’s population reconstructions, the precision was high: 55 to 78 percent of the individuals identified for removal were actually incorrect.

We have also demonstrated an application of allele similarity with our fast SibJoin heuristic. SibJoin is a bottom-up algorithm based on single linkage clustering. Our experiments show that despite being a heuristic, the algorithm competes in accuracy with existing likelihood-based algorithms, but is thousands of times faster in practice. The speed of our algorithm is important since existing algorithms fail to reconstruct half-sibling families when the population size is above a few hundred individuals. SibJoin can reconstruct these populations in seconds. SibJoin is able to reconstruct real biological populations that existing algorithms fail to reconstruct, and it does so with high accuracy.

## Competing interests

The authors declare they have no competing interests.

## Authors’ contributions

DD and DGB jointly developed the algorithms and proofs. DD implemented the algorithms and conducted the experiments. DGB directed the project. Both authors wrote, read, and approve the manuscript.
